# Estimated activity levels in dogs at population scale with linear and causal modeling

**DOI:** 10.3389/fvets.2025.1572794

**Published:** 2025-07-10

**Authors:** Abigail O’Rourke, Richard Haydock, Richard F. Butterwick, Alexander J. German, Aletha Carson, Scott Lyle, Ciaran O’Flynn

**Affiliations:** ^1^Waltham Petcare Science Institute, Waltham on the Wolds, Leicestershire, United Kingdom; ^2^Institute of Life Course and Medical Sciences, University of Liverpool, Neston, United Kingdom; ^3^Pet Insight Project, Whistle Labs, San Francisco, CA, United States; ^4^Machine Intelligence and Decision Systems Research Group, Queen Mary University of London, London, United Kingdom

**Keywords:** dog, canine, accelerometer, activity monitor, wearable technology, real-world

## Abstract

**Introduction:**

The aim of this study was to determine patterns of physical activity in pet dogs using real-world data at a population scale aided by the use of accelerometers and electronic health records (EHRs).

**Methods:**

A directed acyclic graph (DAG) was created to capture background knowledge and causal assumptions related to dog activity, and this was used to identify relevant data sources, which included activity data from commercially available accelerometers, and health and patient metadata from the EHRs. Linear mixed models (LMM) were fitted to the number of active minutes following log-transformation with the fixed effects tested based on the variables of interest and the adjustment sets indicated by the DAG.

**Results:**

Activity was recorded on 8,726,606 days for 28,562 dogs with 136,876 associated EHRs, with the median number of activity records per dog being 162 [interquartile range (IQR) 60–390]. The average recorded activity per day of 51 min was much lower than previous estimates of physical activity, and there was wide variation in activity levels from less than 10 to over 600 min per day. Physical activity decreased with age, an effect that was dependent on breed size, whereby there was a greater decline in activity for age as breed size increased. Activity increased with breed size and owner age independently. Activity also varied independently with sex, location, climate, season and day of the week: males were more active than females, and dogs were more active in rural areas, in hot dry or marine climates, in spring, and on weekends.

**Conclusion:**

Accelerometer-derived activity data gathered from pet dogs living in North America was used to determine associations with both dog and environmental characteristics. Knowledge of these associations could be used to inform daily exercise and caloric requirements for dogs, and how they should be adapted according to individual circumstances.

## 1 Introduction

Both duration and intensity of physical activity affect energy expenditure, and the impact of different activities in dogs has been extensively reviewed (1). However, relatively little is known about physical activity level of pet dogs. Current evidence suggests that many dogs living in single-pet households have relatively sedentary lives (2–4), with a meta-analysis of studies from the USA and Australia indicating a median weekly activity of 4 walks and 160 min (5). However, these data are largely based on owner-reported estimates of physical activity, which are somewhat subjective, open to bias and do not take account of the intensity of activity (6).

Previous studies have found that physical activity in dogs decreases with age (7–10) and potential associations with sex, breed, body mass, neuter status and body condition score are reported (7, 8, 10–12). Environmental factors may also influence physical activity: for example, human routines vary between weekdays and weekends which may impact the activity of companion dogs (13–16). Dogs living in rural environments are reported to be more active, and older owners have been observed to have more active dogs (9).

Objective methods for measuring physical activity, such as heart rate monitoring or accelerometery, have been recommended for studies in humans (17), and have potential application for companion animal research. Previous studies have assessed the validity and reliability of accelerometers for the measurement of physical activity in dogs (18–26). Accelerometers have also been used to assess the effect of clinical treatments (chemotherapy, non-steroidal anti-inflammatory drugs and weight loss) on physical activity in dogs (27–32), to determine correlates of physical activity in common breeds of dog (7, 10, 33, 34), to compare activity patterns in shelters and domestic settings (35, 36), and to evaluate differences in activity between healthy and osteoarthritic dogs (37–40).

Many different activity monitors are now available for dogs, for example those made by Whistle (Mars Petcare, McLean, VA) (41), which provide owners with information in a mobile application regarding the daily activity levels of their dog. The availability and widespread use of such accelerometer technology provides the opportunity to study physical activity in large numbers of dogs kept in a domestic setting.

Studies based on real-world observational data are often limited to identification of associations between variables of interest and the outcome, such that causation cannot be assumed unless a prospective study, such a randomized control trial (RCT), is undertaken. However, both the theory and toolset for making causal inferences from real-world observational data are well-established (42–44), enabling potential biases and confounding, which would be addressed through design and randomization in an RCT. A directed acyclic graph (DAG) is a graphical tool that enables identification of key concepts and assumptions, such as causal pathways and confounders (45, 46), as well as the appropriate adjustment sets for estimating causal effects (47). This approach has been used across many areas of science, including applied healthcare research (48).

The aim of the current study was to examine physical activity patterns in pet dogs, using population-scale data from Whistle accelerometers and electronic health records (EHR), as well as determining causal factors of physical activity in dogs.

## 2 Materials and methods

### 2.1 Data

**Figure 1:**
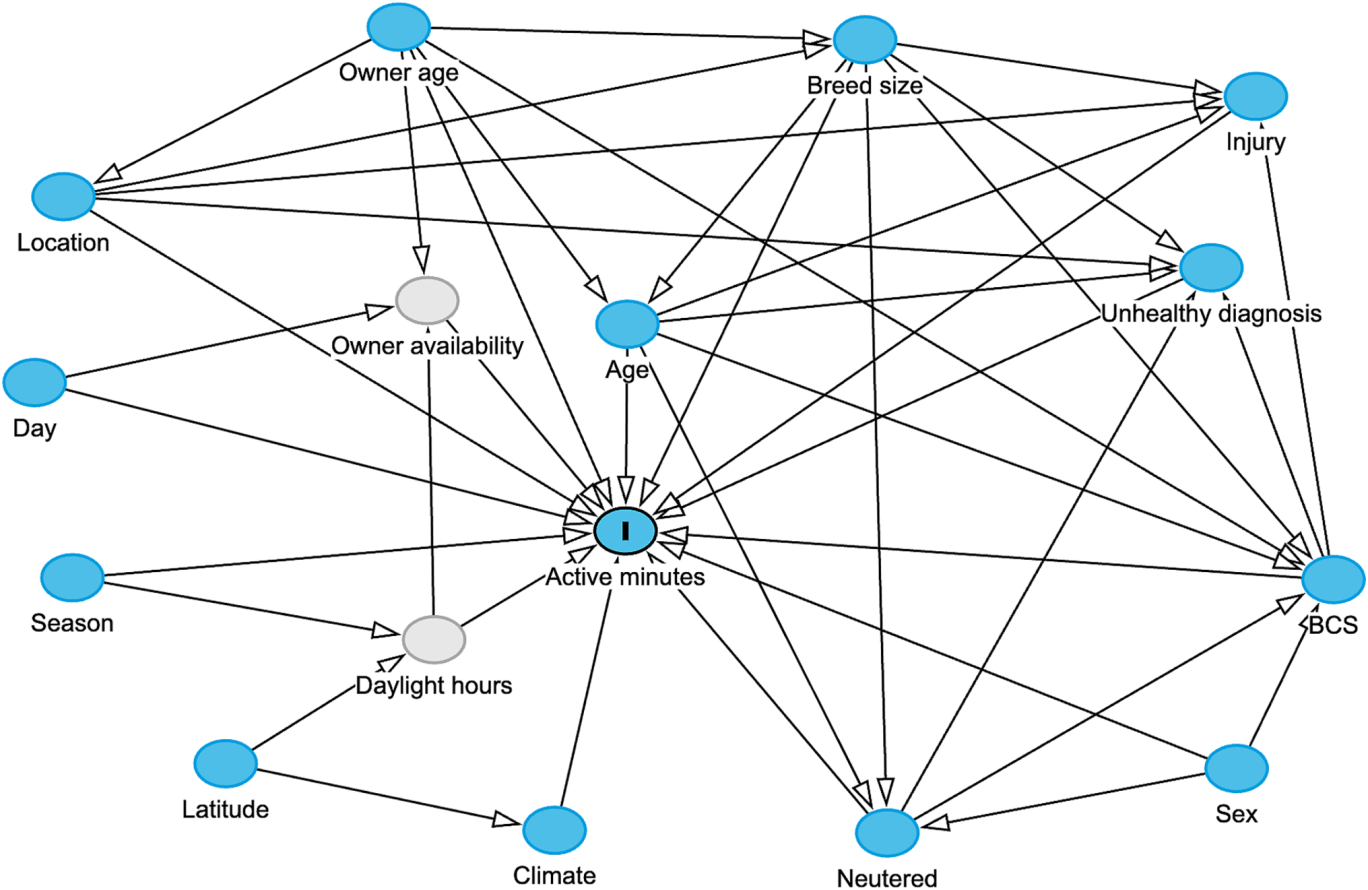
Hypothesized data generating process for activity of an individual dog in a day, displayed in a directed acyclic graph (DAG), created in the web-based application DAGitty (66). Blue circles represent observed variables, grey circles represent unobserved variables, and arrows indicate direction of hypothesized causality. In order to have a causal effect, a causal variables must precede the effect variables in time. For example, “active minutes” is considered at a timepoint subsequent to recording of “unhealthy diagnosis.”

A variety of factors may influence the physical activity levels of a dog including both pet and environmental attributes. Therefore, a DAG was constructed to capture background knowledge and causal assumptions related to dog activity (Figure 1). One of the study authors (AO) constructed the DAG, with input from veterinary experts (AJG), experts in animal nutrition (RFB) and data scientists (COF, RH), further informed by a review of scientific literature. The DAG was created in an iterative process of mapping and integration to identify relevant variables and their putative causal relationships. Relevant data sources were identified based on the DAG: pet and activity data from the Pet Insight Project (49) (a large pet health study that distributed 100,000 activity monitors to clients of Banfield Pet Hospitals to combine activity data from accelerometer devices with EHRs), owner data, and environmental data. Additional details on the hypotheses illustrated in the DAG can be founded in Supplementary Table 1. The timeframe for data inclusion coincided with the timing acquisition of activity records, with the earliest and latest activity records timestamped November 2013 and October 2023.

All internal data (Whistle records, Banfield EHRs and owner data) are anonymized and encrypted with stringent security processes in place, including algorithmic removal of all personally identifiable information. The authors were required to obtain approval from internal data governing bodies to access the anonymized data. Participants of the Pet Insights Project consented to have their pet data utilized for research purposes and were able to opt out at request.

#### 2.1.1 Activity data

Retrospective activity data were extracted by one study author (AO) from an anonymized database of records collected by commercially available accelerometers (Whistle 3, Whistle 4, Whistle FIT, Whistle GO, Whistle Health, Whistle GPS; Mars Petcare) that acquired 3 Hz 3-axis accelerometery data for at least several seconds whenever sufficient movement was detected. The raw data from the accelerometer were converted into the number of minutes per day spent in medium-to-high intensity activity (10, 19). These activity measurements have been validated in laboratory conditions and assessed to be independent of the size of the dog (10). The algorithm also excludes activities from the calculation that do not expend sufficient energy, such as riding in a car, shaking or scratching. All devices measured and processed activity equivalently. Activity records from days where either <10 or >600 active minutes had been recorded were removed (21% of records) because these were likely to be inaccurate measurements, potentially as a result of partial activity monitor use during the day, user error (e.g., an owner leaving a device in their pocket for a prolonged period) or anomalous behavior (e.g., resting after injury or illness).

#### 2.1.2 Electronic health records

Retrospective health data and metadata from canine inpatient visits were extracted by one study author (AO) from the EHR database of Banfield Pet Hospitals (Petware©), a network of over 1,000 primary care veterinary hospitals located primarily in the USA. At the time of registering their pet at the hospital, owners gave consent for retention of anonymised EHR data and its use in clinical studies. Since this study was a secondary analysis of these data, no additional informed consent was requested from the owner. Data were collected between November 2012 and October 2023. The patient information extracted from the EHR database included: breed, date of birth, sex, neuter status, and date of neutering (if available); information extracted for each visit included: body weight (in kg), body condition score (BCS: “1 - very thin,” “2 - thin,” “3 - ideal weight,” “4 - overweight,” and “5 - markedly obese”), diagnoses (e.g., “healthy pet,” “dental calculus,” “dermatitis”) and visit reason (e.g., “comprehensive exam,” “consultation,” “dental cleaning,” “vaccination”). Further details about this EHR database have been described (50).

Patient information is obtained when registering a dog (e.g., date of birth, breed and sex). Birth date might be inaccurately recorded, for example, if the owner does not know the exact date or if it is incorrectly entered into the record. Given that the age (in years) at visit was derived from the date of birth and visit dates, it might also be inaccurate. Neuter status at visit was derived using the neuter status and information about the date of neutering, which is either recorded at registration or when the procedure is undertaken at a Banfield Pet Hospital. If neutering was undertaken elsewhere, the information might not be recorded, or the exact date might not be available. If no date of neutering was available, all visits were considered to have the neuter status given in the patient information. Body weight is routinely recorded at visits using electronic scales, whilst body condition is assessed by a veterinarian either using the 5-point BCS scale (described above) or a 9-point scale which is then translated within the database to the 5-point scale. For the latter, each unit on the 5-point BCS scale corresponded to two points on the 9-point scale (e.g., ideal weight BCS 3/5 vs. 4–5/9; overweight BCS 4/5 vs. 6–7/9; obese 5/5 vs. 8–9/9).

Breed size category was assigned for each dog using previously published thresholds (50); in this respect, adult dogs weighing ≤6.5 kg, 6.5–9.0 kg, 9–15 kg, 15–30 kg, 30–40 kg and >40 kg were assigned to the toy, small, medium-small, medium-large, large and giant category, respectively. To assign a dog to a particular category, the average of all its adult body weight measurements within its EHRs was used, corrected where necessary using BCS; here, the adult weights of dogs recorded as 4/5 and 5/5 were multiplied by 0.8 and 0.6 respectively, to take account of the fact that each unit on the 5-point BCS (or two units on the 9-point BCS) correspond to approximately 20% additional weight (51–54). Following this, a binary “weight status” variable was created comprising dogs recorded as underweight or ideal weight (BCS 1–3/5) or overweight condition (BCS 4–5/5).

The EHRs were classified as healthy or unhealthy based on the reason for the visit and diagnoses recorded at the visit. Injury or ill health is recorded in each EHR until the issue is resolved, not just at the first instance of diagnosis. An EHR was classified as unhealthy if there was a diagnosis (including historic diagnosis) of a chronic illness such as organ failure, any cardiovascular, musculoskeletal, metabolic or endocrine system disorder. A range of different etiologies, including neoplasia, were included within this classification and, when present, the EHR was flagged as “unhealthy visit.” An indicator of potential injury was also added to the EHRs, based on records of ailments or procedures that could affect mobility, such as fractures, road traffic accident, surgery or anesthesia. An EHR was classified as healthy if it was not classed as unhealthy as above, and it was a general preventive care visit, for example, for vaccination, anthelmintic prescription or grooming.

#### 2.1.3 Environmental and owner data

Additional data on location and owner age group were extracted from the Banfield EHRs and validated by using data provided by Epsilon (55) as a secondary source. Location data were summarized into three variables: climate zone, latitude and location type. For the climate zone variable, dogs were assigned to one of 4 categories (cold, hot-humid, hot-dry and marine), which were simplified categories based on the zones used in climate-specific guidance from the Building America project (56); the location type variable was classified as urban or rural, based estimates of population density by census tract: locations were assigned to tracts, counties and states using data from the US Census Bureau via the open data FTP (57). Population density and rurality group data were retrieved from the US Department of Agriculture (58).

Linking activity and EHR data (see below) enabled variables to be created for season (spring, summer, autumn, winter) and day type (weekday, weekend). The season variable was based on northern hemisphere dates for meteorological seasons (59) assigned using the calendar day on which the activity was recorded, whilst the day type variable was based on the day of the week that the activity occurred (weekday: Monday to Friday; weekend: Saturday and Sunday).

### 2.2 Joining dog, owner, household, and activity data

**Figure 2:**
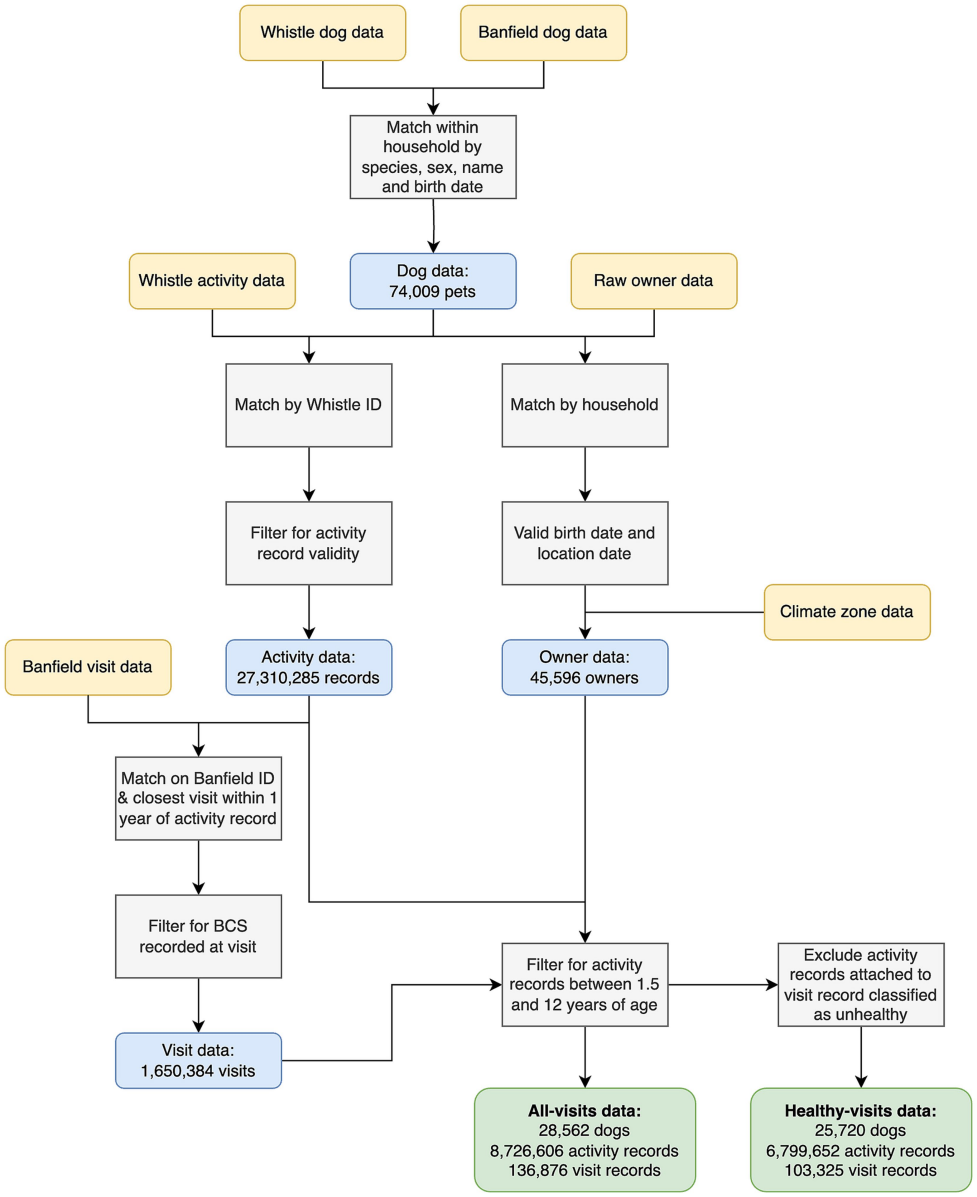
Flow diagram illustrating the steps used in data cleaning and generation of the final study data. Yellow boxes depict the datasets used, grey boxes depict the actions undertaken, blue boxes depict the data available at each stage, whilst the green boxes show the final “all-visits” and “healthy-visits” datasets. “Whistle” refers to the data from the accelerometer, including dog demographic data and daily activity records. “Banfield” refers to the electronic health record data, including dog and anonymized owner demographic data, and visit data.

Full details of how data derived from the activity monitors and EHRs were linked are shown in Figure 2. Briefly, data from each source were matched within household, by species, sex, name and date of birth (month and year). Matching the data between the two sources in this way enhances reliability; for example, finding that the same date of birth is recorded in both the activity and EHR databases, increases confidence that the data are reliable. The matching was performed algorithmically, via a graph network on anonymized data, and a linking identifier was provided to researchers to enable the datasets to be joined. Data on owner city, county and state were used to match the location and climate zone data. To join dog data, such as health and weight status to activity, each daily activity record for each dog was linked to the closest veterinary visit in the EHR, provided that the visit occurred before the activity record and within one calendar year. This results in a one-to-many relationship for visits to activity records, in that the dog data from a visit record may be joined to multiple activity records following it. Activity records that were not within 1 year of a veterinary visit were excluded from analysis. The activity records were linked to visit records regardless of whether they were classified as healthy, such that filtering could be performed later to remove activity records nearby visits categorized as unhealthy. The ratio of activity records to visit records was inspected to identify extreme outliers. A threshold of 5:1 was determined based on visual inspection; dogs with fewer than five activity records per visit were excluded. These were often dogs with high visit frequencies and low numbers of activity records.

The final inclusion criteria were determined by a combination of opinion from veterinary experts (AJG) and data scientists (AO, COF, RH), the quantity and quality of data and the relevance of the data to the aims of the study. For inclusion in the final datasets, dogs had to have more than seven activity records available, to avoid sparsity in the data at a dog level. Further, they had to be >1.5 years but <12 years of age at both the time of activity record and linked visit record, to ensure that they were adult at the time the data were recorded. The “all visits dataset” comprised data from EHRs regardless of classification (healthy vs. unhealthy). However, to examine associations between dog variables studied and activity of dogs without any illness or injury, a “healthy visits dataset” was also created (Supplementary Table 2), by removing activity records linked to EHRs with a record of either an injury or unhealthy diagnosis.

### 2.3 Statistical analysis of active minutes

Statistical analysis was performed using an online open-access statistical language and environment (R version 4.2.3) (60), with several additional packages: *dotwhisker* v0.7.4 (61), *ggeffects* v1.3.0 (62), *ggplot2* v3.4.3 (63), *lmerTest* v3.1–3 (64), *dbarts* v0.9–23 (65). The DAG was tested for consistency with the data by checking the suggested conditional independences (see Supplementary material for more details). The candidate independent variables were checked for multicollinearity using Pearson’s correlation coefficient for continuous variables and Cramer’s V for categorical variables, where values above 0.5 would be require further consideration. Linear mixed models (LMM) were fitted to the number of active minutes following log-transformation using the *lmer* function (*lmerTest*) (64) whereby 30 activity records per dog were selected at random and used in the analysis. Fixed effects were identified based on the variables of interest and the adjustment sets indicated by the DAG using the DAGitty software v3.1 (66). The random effect was specified as an intercept per dog to account for repeated measures within the data. Interactions were considered if they were hypothetically plausible and were assessed by comparing the Akaike information criteria (AIC) (67) of models with and without the interaction terms, determined by the *anova* function. To balance complexity against performance, interactions were only included if they resulted in a statistically-significant (*p* < 0.05) reduction in AIC.

To validate the models, the dataset being analyzed was subdivided into five samples, with records grouped by dog ensuring that all records for an individual dog were placed into the same sample. A LMM as described above was fitted to each data subset and the coefficients and performance across the remaining four subsets were compared for consistency. The final results for the LMM were generated using a permutation testing strategy, whereby the process of random data sampling of 50 activity records per dog and model fitting was repeated 1,000 times. The coefficient estimates and confidence intervals were determined as the mean of the 1,000 individual models. The *p*-values for the coefficient estimates were calculated as the fraction of time the confidence interval included zero. This approach produces coefficient estimates and p-values less susceptible to noise in the data sample.

Further model validation, including verification of the adjustment set indicated by the DAG, was conducted by training a non-parametric Bayesian additive regression trees (BART) model (68). The BART model should be insensitive to confounders, meaning that it is not necessary to identify the correct adjustment set. The model was fitted to the log-transformed number of active minutes, with all predictors present in the DAG and dog ID as the grouping factor, using the *rbart*_vi function (*dbarts*) (65). The correlation of predictions and partial dependence plots from the LMM and BART models were compared for consistency.

## 3 Results

### 3.1 Study population

**Figure 3:**
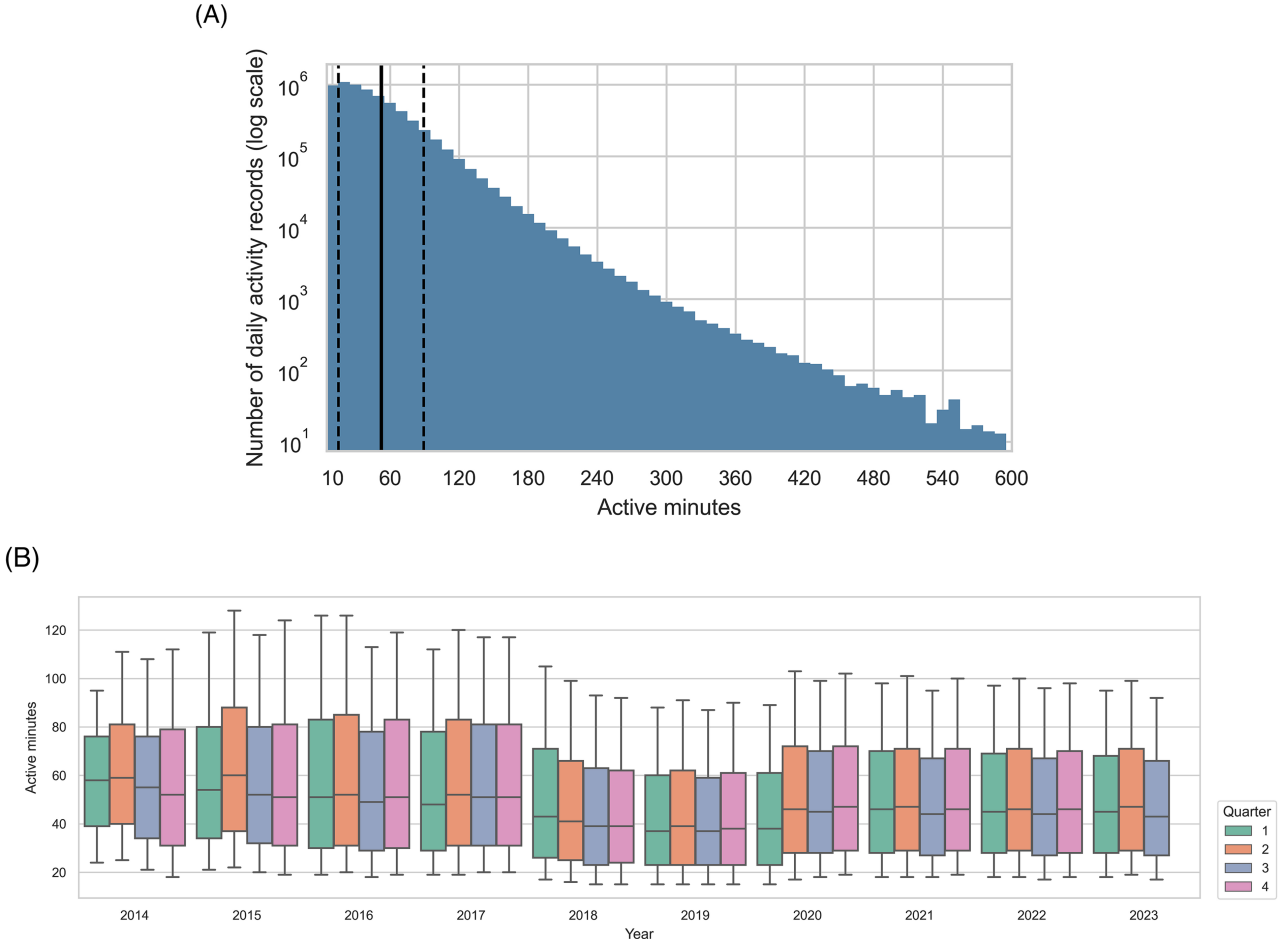
Summarization of active minutes within the all-visits dataset. **(A)** Distribution of active minutes recorded per activity record in the study population, with number of records on a log scale. The bin width is 10 min, the solid vertical line shows the mean value, and the dashed vertical lines show one standard deviation from the mean. **(B)** Active minutes recorded over the data collection period for the study. The upper and lower limits of the boxes show 25th and 75th percentiles, whilst bold horizontal line within each box show the 50th percentile. Given that minimum and maximum values were outside the thresholds set during the selection process, the whiskers show the 10th and 90th percentiles.

The all-visits dataset comprised activity recorded 8,726,606 days for 28,562 dogs with 136,876 associated EHRs, and the healthy visits dataset contained 6,799,652 days of activity records for 25,720 dogs with 103,325 EHRs. The mean number of activity records per dog was 305.5 [standard deviation (SD) 372.20, median 162, interquartile range (IQR) 60–390] in the all-visits dataset, and 322.3 (SD 383.2, median 174, IQR 65–419) in the healthy-visits dataset. Figure 3A illustrates the association between the number of activity records included and number of active minutes per record for the all-visits dataset. The mean number of active minutes per dog was 50.8 (SD 36.05) minutes per day in the all-visits dataset and 52.3 (SD 36.95) in the healthy-visits dataset. The distribution of active minutes recorded over the data collection period was stable, as shown in Figure 3B for the all-visits dataset.

A summary of dog, activity and visit data stratified by breed size for the study population (all-visits) compared with that of the Banfield population is shown in Table 1. There were 15,016 (52.6%) male and 13,546 (47.4%) female dogs, with the mean age at time of recording activity being 6.1 (SD 2.74) years. Of the 136,876 visits, 131,140 (95.9%) and 46,820 (34.2%) involved dogs reported to be neutered or in overweight condition, respectively. Dogs of 225 different breeds were represented (Supplementary Table 2 presents the 30 most common breeds), with dogs of the Labrador Retriever breed (purebred or mix) being most common (3,295 dogs; 11.5% of all dogs and activity records). For comparison, 441,812 (9.0%) and 10,424 (5.8%) Labrador retrievers (purebred or mix) visited Banfield Veterinary Hospitals or were registered with Whistle within the same timeframe, respectively. Dogs labeled as “Mixed Breed” were next most common (2,312 dogs, 8.1% of all dogs and records), which was similar to the proportion of “Mixed Breed” dogs in the Banfield (386,081 dogs, 7.9%; 2nd most common) and Whistle (3,311 dogs, 1.9%) populations. Conversely, Chihuahua (purebred or mixed) was the third most prevalent named breed in the Banfield population (8.1%, 394,416 dogs) but represented only 5.1% (1,460 dogs) in the study dataset and 2.0% (3,512 dogs) in the Whistle population.

**Table 1:** Summary of the datasets used in the study stratified by breed size and compared with the Banfield population to evaluate potential selection bias.

Variable and dataset	Breed size						
	All dogs	Toy^1^	Small^1^	Medium-small^1^	Medium-large^1^	Large^1^	Giant^1^
Dogs^1^							
All-visits population	28,562	5,095 (17.8%)	3,305 (11.6%)	3,760 (13.2%)	10,631 (37.2%)	4,612 (16.2%)	1,159 (4.1%)
Healthy-visits population	25,720	4,191 (16.3%)	2,915 (11.3%)	3,441 (13.3%)	9,115 (38.5%)	4,232 (16.5%)	1,056 (4.1%)
Banfield population^2^	4,901,786	1,401,458 (28.6%)	625,138 (12.8%)	578,338 (11.8%)	1,503,240 (30.7%)	636,219 (13.0%)	156,393 (3.2%)
Activity records^1^							
All-visits population	8,726,606	1,466,824 (16.8%)	1,077,408 (12.4%)	1,246,939 (14.3%)	3,254,134 (37.3%)	1,383,940 (15.9%)	297,361 (3.4%)
Healthy-visits population	6,799,652	945,986 (13.9%)	775,495 (11.4%)	990,760 (14.6%)	2,738,039 (40.3%)	1,113,763 (16.4%)	235,609 (3.5%)
Activity records per dog							
All-visits population	305.5 (372.20)	287.9 (362.01)	326.0 (392.2)	331.6 (393.79)	306.10 (373.74)	300.1 (356.24)	256.6 (321.35)
Healthy-visits population	322.3 (383.20)	316.4 (389.75)	347.4 (406.10)	348.7 (404.67)	317.9 (381.65)	313.9 (365.29)	267.5 (328.14)
Active minutes per day							
All-visits population	50.8 (36.05)	61.8 (44.20)	62.7 (45.18)	67.9 (48.24)	71.5 (53.56)	71.5 (52.09)	65.5 (48.15)
Healthy visits population^2^	52.3 (36.95)	61.1 (43.01)	62.6 (44.92)	68.3 (48.05)	71.6 (53.43)	72.0 (52.11)	66.1 (48.50)
Visits^1^							
All-visits population	136,876	23,379 (17.1%)	17,157 (12.5%)	19,676 (14.4%)	50,053 (36.6%)	21,767 (15.9%)	4,844 (3.5%)
Healthy-visits population	103,325	14,683 (14.2%)	11,986 (11.6%)	15,056 (14.6%)	40,975 (39.6%)	16,894 (16.4%)	3,731 (3.6%)
Banfield population^2^	60,105,063	17,595,443 (29.3%)	8,367,457 (13.9%)	7,603,311 (12.7%)	17,163,959 (28.6%)	7,621,122 (12.7%)	1,753,760 (2.9%)
Visits per year							
All-visits population	3.1 (1.85)	3.0 (1.69)	3.3 (1.89)	3.4 (2.20)	3.1 (1.82)	3.1 (1.72)	2.9 (1.50)
Healthy-visits population	3.0 (1.74)	2.9 (1.60)	3.1 (1.72)	3.2 (2.01)	3.0 (1.73)	3.0 (1.65)	2.8 (1.50)
Banfield population^2^	3.4 (1.68)	3.3 (1.65)	3.5 (1.75)	3.6 (1.85)	3.3 (1.59)	3.4 (1.65)	3.5 (1.68)
Age at activity record (years)							
All-visits population	6.1 (2.75)	6.7 (2.79)	6.7 (2.77)	6.2 (2.82)	5.7 (2.66)	5.8 (2.66)	5.4 (2.50)
Healthy-visits population	5.6 (2.59)	6.2 (2.71)	6.3 (2.69)	5.8 (2.69)	5.4 (2.48)	5.4 (2.46)	5.0 (2.34)
Age at visit record (years)							
All-visits population	6.1 (2.83)	6.7 (2.85)	6.8 (2.82)	6.3 (2.89)	5.7 (2.75)	5.8 (2.75)	5.3 (2.55)
Healthy-visits population	5.7 (2.67)	6.3 (2.8)	6.3 (2.73)	5.9 (2.79)	5.4 (2.57)	5.3 (2.54)	4.9 (2.36)
Banfield population^2^	5.6 (3.88)	6.3 (4.01)	6.4 (4.02)	5.9 (3.98)	5.0 (3.64)	4.9 (3.53)	4.3 (3.21)
Body weight at visit (kg)							
All-visits population	20.9 (13.30)	5.2 (1.44)	8.4 (1.41)	12.8 (2.68)	25.5 (5.62)	37.4 (5.29)	51.8 (9.64)
Healthy-visits population	21.6 (13.02)	5.3 (1.41)	8.3 (1.41)	12.8 (2.72)	25.4 (5.56)	37.2 (5.32)	51.6 (9.64)
Banfield population^2^	16.5 (12.96)	4.6 (1.74)	7.9 (1.92)	11.9 (3.30)	23.9 (7.13)	32.9 (9.08)	43.9 (14.34)
Male sex ^3^							
All-visits population	15,016 (52.6%)	2,540 (49.9%)	1,945 (58.9%)	2,089 (55.6%)	4,561 (42.9%)	3,052 (66.2%)	829 (71.5%)
Healthy-visits population	13,533 (52.6%)	2,087 (49.8%)	1,715 (58.8%)	1,886 (55.3%)	4,268 (43.1%)	2,817 (66.6%)	760 (72.0%)
Banfield population^2^	2,66,635 (52.6%)	609,676 (46.8%)	361,769 (58.8%)	344,741 (58.1%)	597,716 (43.5%)	446,820 (66.2%)	164,665 (75.4%)
Visits recording neutered status^3,4^							
All-visits population	131,140 (95.9%)	21,730 (93.0%)	16,518 (96.4%)	19,074 (97.0%)	48,722 (97.4%)	20,662 (95.0%)	3,495 (91.6%)
Healthy-visits population	99,029 (95.9%)	13,643 (93.0%)	11,544 (96.4%)	14,597 (97.0%)	39,843 (97.3%)	15,981 (94.7%)	3,421 (91.7%)
Banfield population^2^	53,748,471 (89.4%)	15,148,341 (86.1%)	7,661,236 (91.6%)	7,009,379 (92.2%)	15,726,785 (91.6%)	3,287,840 (88.7%)	1,444,566 (82.4%)
Visits recording overweight condition^3,4^							
All-visits population	46,820 (34.2%)	6,057 (25.9%)	6,382 (37.2%)	7,691 (39.1%)	16,369 (32.7%)	8,558 (39.4%)	4,613 (36.4%)
Healthy-visits population	33,504 (32.5%)	3,345 (22.8%)	4,133 (34.5%)	5,632 (37.4%)	12,776 (31.2%)	6,303 (37.3%)	1,315 (35.3%)
Banfield population^2^	17,767,362 (29.6%)	4,083,723 (23.2%)	2,799,537 (33.5%)	2,727,957 (35.9%)	5,166,712 (30.1%)	2,494,825 (32.7%)	494,608 (28.2%)

Results of continuous datasets are reported as mean (standard deviation), whilst counts and categorical data are reported as number (%). ^1^For dogs, activity records and visits, the percentages shown for breed groups represent the percentage of that breed group within the all-dog population. ^2^The Banfield population is defined as dogs visiting Banfield Pet Hospitals between in the years 2013 to 2023, to align with the data collection period for the study. ^3^For sex, neuter status and overweight condition, percentages represent percentage of male dogs, neutered dogs and overweight dogs, respectively, within the all-dog population or respective breed group. ^4^Given that both neuter status and overweight status may change over the data collection period, these numbers are reported relative to visits rather than individual dogs.

### 3.2 Associations between dog attributes and physical activity in dogs

Based on the DAG (Figure 1), adjustments for possible effects of location and owner age would be necessary to examine associations between activity and dog variables (age, breed, size, weight status, sex and neuter status). Further, it was not necessary to include either the unhealthy diagnosis or injury variables, because the DAG indicated that these were modifiers of other effects (e.g., age, breed size, BCS and neuter status). A linear-mixed effects model was created to determine associations between log-transformed active minutes and dog age, breed size, weight status, sex, neuter status, owner age and location. In initial models, the following plausible interactions were also assessed: age and breed size, weight status, neuter status, owner age; size and weight status; breed size and owner age; weight status and neuter status; neuter status and sex. However, the only significant interaction identified, and therefore, included in the final model, was the interaction between breed size and age (Table 2). This model had an R^2^ of 0.515 and a root square mean deviation (RMSE) of 0.462, whilst the random effect (pet ID) accounted for 0.221 of the variance in the data. Model coefficients and performance were consistent across each of five data subsets (SD for R^2^ 0.0108; SD for RMSE 0.0154). The trends observed in the final model were consistent with those observed in the non-parametric BART model for each attribute. Additional model performance metrics are reported in Table 2.

**Table 2:** Linear mixed-effects model of log-transformed activity as a function of dog-related variables, whilst controlling for owner age and location (urban or rural), using the final dataset.

Variables	Estimate	Error	2.5% CL	97.5% CL	*p*-value	Marginal mean (LM)	2.5% CL	97.5% CL	Marginal mean (BART)	2.5% CL	97.5% CL
Intercept	34.866	0.017	33.689	36.083	< 0.001	–	–	–	–	–	–
Dog age^−2^	2.432	0.020	2.303	2.565	< 0.001	–	–	–	–	–	–
Owner age	0.033	0.002	0.028	0.037	< 0.001	–	–	–	–	–	–
Breed size											
Toy	−0.242	0.014	−0.263	−0.221	< 0.001	37.0	36.4	37.6	39.4	38.2	40.3
Small	−0.202	0.016	−0.227	−0.176	< 0.001	37.7	37.0	38.5	41.9	40.6	43.2
Medium-small	−0.051	0.015	−0.077	−0.023	0.001	40.4	39.6	41.2	42.6	41.4	43.7
Medium-large	Reference category					40.3	39.7	40.9	41.7	41.0	42.5
Large	0.054	0.014	0.026	0.083	< 0.001	41.2	40.4	41.9	43.6	42.5	44.8
Giant	−0.031	0.024	−0.076	0.015	0.872	35.5	34.4	36.6	38.0	36.4	39.7
Weight status											
Ideal weight	Reference category					38.7	38.1	39.2	42.0	41.6	42.5
Overweight	−0.002	0.002	−0.005	0.002	0.896	38.6	38.1	39.1	40.7	40.1	41.3
Neuter status											
Intact	Reference category					39.0	38.0	39.9	42.6	40.4	44.4
Neutered	−0.017	0.013	−0.042	0.008	1.000	38.3	38.0	38.6	41.6	41.1	42.0
Sex											
Male	Reference category					39.5	39.0	40.1	42.5	41.9	43.2
Female	−0.045	0.006	−0.056	−0.034	< 0.001	37.8	37.2	38.3	40.6	39.9	41.1
Location											
Rural	Reference category					–	–	–	–	–	–
Urban	−0.004	0.006	−0.015	0.008	1.000	–	–	–	–	–	–
Breed size-age interaction											
Toy	0.010	0.002	0.007	0.014	< 0.001	–	–	–	–	–	–
Small	0.005	0.002	0.001	0.008	0.241	–	–	–	–	–	–
Medium-small	−0.013	0.002	−0.017	−0.010	< 0.001	–	–	–	–	–	–
Medium-large	−0.023	0.001	−0.025	−0.020	< 0.001	–	–	–	–	–	–
Large	−0.028	0.002	−0.031	−0.025	< 0.001	–	–	–	–	–	–
Giant	−0.039	0.002	−0.046	−0.032	< 0.001	–	–	–	–	–	–

The details include intercept, fixed effects coefficients and their 2.5 and 97.5% confidence limit (CL) and *p*-values. Estimated marginal mean activity is displayed for values of categorical variables for the linear model (LM) and the BART model, where all other variables are adjusted for mean values (dog age of 6.0 years and owner age of 41.7 years). The model had an R^2^ of 0.515 (SD 0.0108) and a RMSE of 0.462 (SD 0.0154) on log-transformed active minutes, whilst pet ID as a random effect accounted for 0.221 of the variance in the data.

**Figure 4:**
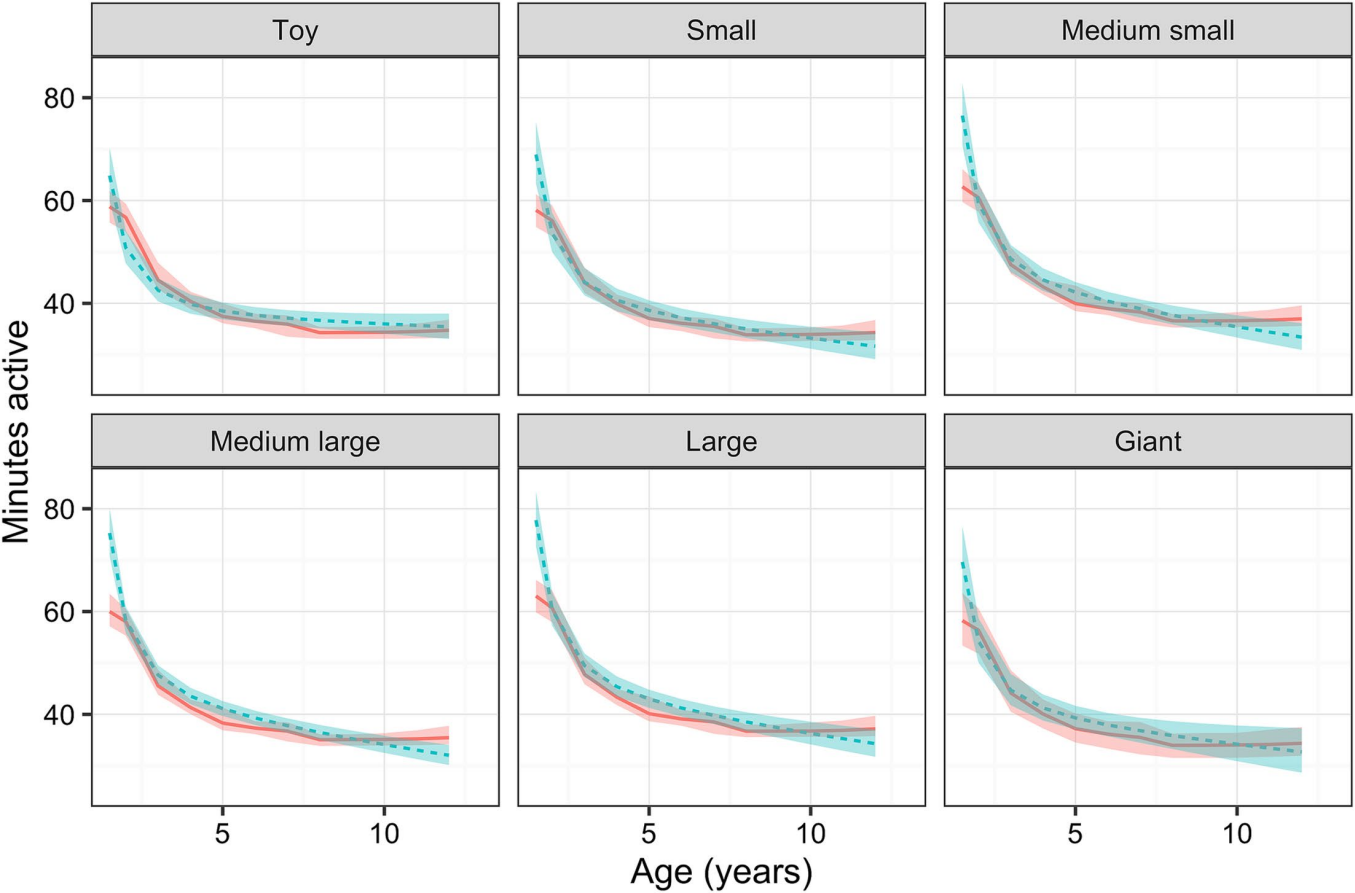
Relationship between active minutes as a function of age for dogs of each breed size group, whilst holding all other relevant variables (body condition score, neuter status, sex, location and owner age) constant, estimated using a linear mixed-effects (LM; blue) and Bayesian additive regression trees (BART; red) models. The ribbons show either the 95% confidence intervals or 95% credible intervals for of those predictions (LM and BART, respectively).

Physical activity decreased with age, an effect that was dependent on breed size, with a greater decline in activity for age observed as breed size increased (Figure 4). For a medium-large dog (reference size), the predicted marginal mean activity for a 1.5-year-old dog was 68.6 active minutes [95% confidence interval (95%-CI) 67.4, 69.9], decreasing to 39.5 active minutes (39.0, 40.1) and 35.4 active minutes (34.8, 35.9) for a 5- and 10-year-old dog, respectively. Activity also varied independently with breed size (*p* < 0.001): for a 6-year-old dog (population mean), the predicted marginal mean activity was 37.0 min (36.6, 37.9) for toy breeds, 37.7 (37.0, 38.5) for small breeds, 40.4 (39.6, 41.2) for medium-small breeds, 40.3 (39.7, 40.9) for medium-large breeds and 41.2 (40.4, 41.9) for large breeds. The predicted marginal mean activity was 35.5 (34.4, 36.6) for giant breeds with a *p*-value of 0.872. Male dogs [39.5 active minutes (39.0, 40.1)] were estimated to be slightly more active (*p* < 0.001) than female dogs [37.8 active minutes (37.2, 38.3)]; however, there was no effect of either weight or neuter status on activity.

Given uncertainties about possible effects of illness and injury on physical activity, associations between physical activity and dog attributes were also estimated using the healthy-visits dataset. For this, a linear-mixed effects model was trained as described for the all-visits dataset, again using log-transformed active minutes as the outcome variable and the same predictor variables (and interactions). The final model had an R^2^ of 0.519 and a root square mean deviation (RMSE) of 0.461, whilst the random effect (pet ID) accounted for 0.214 of the variance in the data (Table 3). Model coefficients and performance were consistent across each of five subsamples of the data (SD for R^2^ 0.0112; SD for RMSE 0.0037). The trends observed in the final model were consistent with those observed in the non-parametric BART model for each attribute. The results of the final model were also consistent with those of the model from the all-visits dataset (Table 3).

**Table 3:** Linear mixed-effects model of log-transformed activity as a function of dog-related variables, whilst controlling for owner age and location (urban or rural), using the healthy visit dataset.

Variables	Estimate	Error	2.5% CL	97.5% CL	*p*-value	Marginal mean (LM)	2.5% CL	97.5% CL	Marginal mean (BART)	2.5% CL	97.5% CL
Intercept	35.622	0.018	34.338	36.952	< 0.001	–	–	–	–	–	–
Age^−2^	2.308	0.021	2.178	2.445	< 0.001	–	–	–	–	–	–
Owner age	0.034	0.002	0.029	0.039	< 0.001	–	–	–	–	–	–
Breed size											
Toy	−0.242	0.016	−0.264	−0.218	< 0.001	37.8	37.1	38.5	40.3	39.2	41.5
Small	−0.199	0.018	−0.225	−0.171	< 0.001	38.5	37.6	39.3	40.5	39.2	41.9
Medium-small	−0.061	0.016	−0.089	−0.032	< 0.001	41.4	40.6	42.3	43.8	42.6	45.1
Medium-large	Reference category					41.0	40.4	42.7	42.9	42.0	43.6
Large	0.049	0.014	0.020	0.079	0.005	41.9	41.2	42.8	42.9	41.9	44.1
Giant	−0.014	0.026	−0.062	0.037	0.996	36.2	35.0	37.4	40.0	38.1	41.7
Weight status											
Ideal weight	Reference category					39.5	38.9	40.1	42.4	41.9	42.9
Overweight	−0.004	0.002	−0.008	0.000	0.481	39.3	38.8	39.9	41.8	41.2	42.5
Neuter status											
Entire	Reference category					39.8	38.8	40.9	42.3	40.4	43.9
Neutered	−0.019	0.014	−0.045	0.008	1.000	39.0	38.7	39.4	42.3	41.8	42.8
Sex											
Male	Reference category					40.3	39.7	40.9	43.2	42.4	43.8
Female	−0.043	0.006	−0.054	−0.031	< 0.001	38.6	38.0	39.2	41.3	40.6	42.0
Location											
Rural	Reference category					–	–	–	–	–	–
Urban	−0.004	0.006	−0.016	0.008	1.000	–	–	–	–	–	–
Breed size-age interaction											
Toy	0.010	0.002	0.006	0.014	< 0.001	–	–	–	–	–	–
Small	0.003	0.002	−0.001	0.007	0.833	–	–	–	–	–	–
Medium-small	−0.013	0.002	−0.016	−0.009	< 0.001	–	–	–	–	–	–
Medium-large	−0.026	0.001	−0.028	−0.023	< 0.001	–	–	–	–	–	–
Large	0.049	0.002	−0.034	−0.026	< 0.001	–	–	–	–	–	–
Giant	−0.045	0.004	−0.062	−0.037	< 0.001	–	–	–	–	–	–

The details include intercept, fixed effects coefficients and their 2.5 and 97.5% confidence limits (CL) and *p*-values. Estimated marginal mean activity is displayed for values of categorical variables for the linear model (LM) and the BART model, where all other variables are adjusted for mean values (dog age of 6.0 years and owner age of 41.7 years). The model had an R^2^ of 0.528 and a RMSE of 0.461 on log-transformed active minutes, whilst pet ID as a random effect accounted for 0.214 of the variance in the data.

### 3.3 Association of environmental variables with physical activity in dogs

Possible associations between activity and season, climate, latitude, location type (urban or rural), day type (weekday or weekend) and owner age were examined by the DAG, with no additional confounders indicated (Figure 1). The absence of confounding from dog attributes meant that it was only necessary to perform the analyses on the all-visits dataset. Therefore, a linear-mixed effects model was trained on log-transformed active minutes as a function of season, climate, latitude, location type, day type and owner age, with pet ID as a random effect to account for repeated measures. All plausible interactions were assessed: climate and season; day and season, location, owner age. Significant interactions were identified between climate and season, and between owner age and day. The final model had an R^2^ of 0.533 and a RMSE of 0.447 on log-transformed active minutes, with the random effect (pet ID) accounting for 0.246 of variance in the data. Model coefficients and performance were again consistent across each of five subsamples of the data (SD for R^2^ 0.0105; SD for RMSE 0.0023). Once again, the trends observed in the final model were consistent with those observed in the non-parametric BART model for each attribute.

**Figure 5:**
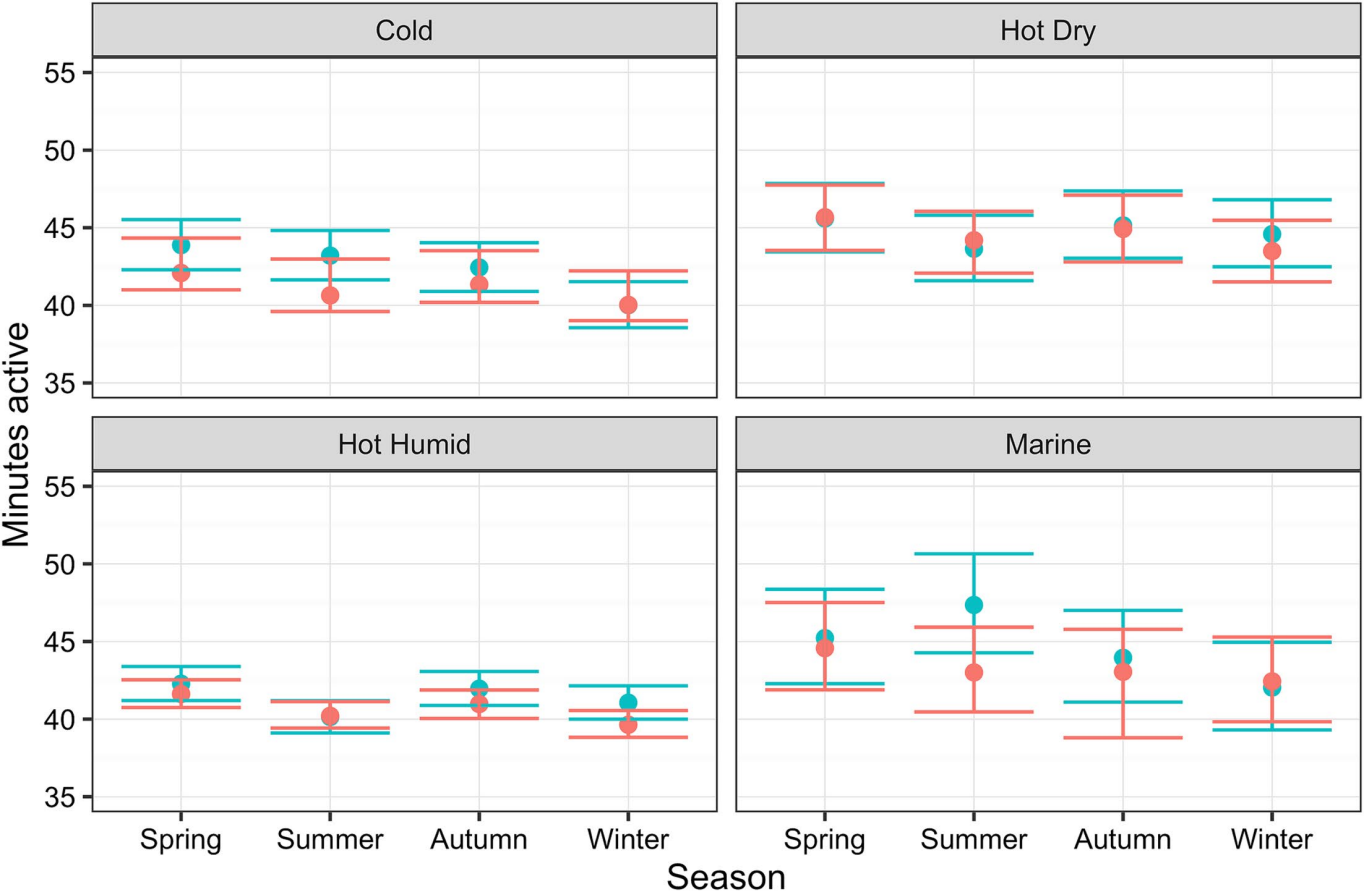
Relationship between active minutes as a function of season and climate, whilst holding all other relevant variables (location, latitude, owner age and day type) constant, estimated using linear mixed-effects (LM; blue) and Bayesian additive regression trees (BART; red) models. The ribbons show either the 95% confidence intervals or 95% credible intervals for of those predictions (LM and BART, respectively).

Estimated marginal means for each environmental attribute are displayed in Table 4. Physical activity was estimated to vary with climate (*p* < 0.001); compared with cold climate as the reference category [41.8 active minutes (41.2, 42.4)], activity levels were greater in dogs living in hot dry [44.2 active minutes (43.4, 45.0)] or marine [44.6 active minutes (43.5, 45.7)] climates, but not in those living in hot humid [41.6 active minutes (41.2, 42.0)] climates. Regarding season, compared with spring as the reference category spring [44.1 active minutes (43.7, 44.5)], dogs were less active in winter [41.6 active minutes (41.2, 42.0)]. Differences between reference and autumn or summer were less significant. Figure 5 shows the predicted activity varying by climate and season, for both the LM and BART predictions, where seasonal trends appear to vary by climate.

**Table 4:** Linear mixed-effects model of log-transformed activity as a function of owner and environmental variables using the all-visits dataset.

Variables	Estimate	Error	2.5% CL	97.5% CL	*p*-value	Marginal mean (LM)	2.5% CL	97.5% CL	Marginal mean (BART)	2.5% CL	97.5% CL
Intercept	28.650	0.038	26.581	30.874	< 0.001	–	–	–	–	–	–
Latitude	0.072	0.009	0.054	0.090	< 0.001	–	–	–	–	–	–
Climate											
Cold	Reference category					41.8	41.2	42.4	40.4	40.0	41.8
Hot humid	−0.006	0.010	−0.026	0.014	1.000	41.5	41.1	41.9	41.3	40.6	42.2
Hot dry	0.060	0.013	0.035	0.087	< 0.001	44.2	43.5	45.0	44.1	42.8	45.7
Marine	0.061	0.014	0.034	0.090	< 0.001	44.6	43.5	45.7	44.3	41.7	47.1
Season											
Spring	Reference category					44.1	43.7	44.5	42.7	42.2	43.4
Summer	−0.009	0.003	−0.014	−0.003	0.083	43.0	42.6	43.4	40.2	39.7	40.8
Autumn	−0.012	0.003	−0.017	−0.006	0.004	43.3	42.9	43.7	41.7	41.1	42.3
Winter	−0.082	0.003	−0.087	−0.077	< 0.001	41.6	41.2	42.0	41.8	41.2	42.4
Day											
Weekday	Reference category					41.3	41.0	41.7	40.6	40.2	41.1
Weekend	0.114	0.002	0.110	0.119	< 0.001	44.7	44.3	45.1	44.1	43.6	44.6
Location											
Rural	Reference category					43.6	43.1	44.1	42.6	41.9	43.3
Urban	−0.027	0.006	−0.038	−0.014	< 0.001	42.4	42.0	42.8	41.9	41.2	42.6
Owner age											
18–30	Reference category					44.6	44.1	45.1	37.8	39.0	39.8
30–45	−0.031	0.005	−0.040	−0.022	< 0.001	42.8	42.4	43.2	38.2	39.1	40.9
45–60	−0.027	0.006	−0.039	−0.016	< 0.001	42.7	42.3	43.2	39.9	41.1	42.3
60+	−0.029	0.008	−0.044	−0.014	0.002	41.9	41.3	42.5	42.8	44.7	46.6
Day-owner age interaction; day = weekend (reference category = weekday)											
18–30	Reference category					–	–	–	–	–	–
30–45	−0.018	0.003	−0.023	−0.0013	< 0.001	–	–	–	–	–	–
45–60	−0.030	0.003	−0.036	−0.025	< 0.001	–	–	–	–	–	–
60+	−0.064	0.004	−0.070	−0.057	< 0.001	–	–	–	–	–	–
Season-climate interaction (season reference category = spring; climate reference category = cold)											
Summer: hot humid	−0.039	0.003	−0.046	−0.033	< 0.001	–	–	–	–	–	–
Summer: hot dry	−0.047	0.005	−0.056	−0.038	< 0.001	–	–	–	–	–	–
Summer: marine	0.025	0.006	0.013	0.037	0.003	–	–	–	–	–	–
Autumn: hot humid	−0.004	0.004	−0.011	0.003	0.850	–	–	–	–	–	–
Autumn: hot dry	−0.012	0.005	−0.022	0.003	0.249	–	–	–	–	–	–
Autumn: marine	−0.010	0.006	−0.022	0.002	0.612	–	–	–	–	–	–
Winter: hot humid	0.043	0.003	0.036	0.050	< 0.001	–	–	–	–	–	–
Winter: hot dry	0.059	0.005	0.049	0.070	< 0.001	–	–	–	–	–	–
Winter: marine	0.010	0.006	−0.002	0.002	0.647	–	–	–	–	–	–

The marginal means for the linear model (LM) and the BART model are displayed. The details include intercept, fixed effects coefficients and their 2.5 and 97.5% confidence limits (CL) and *p*-values. Estimated marginal mean activity is displayed for values of categorical variables, where all other variables are adjusted for mean values (dog age of 6.0 years and owner age of 41.7 years). The model had an R^2^ of 0.533 and a RMSE of 0.447 on log-transformed active minutes, whilst pet ID as a random effect accounted for 0.246 of the variance in the data.

**Figure 6:**
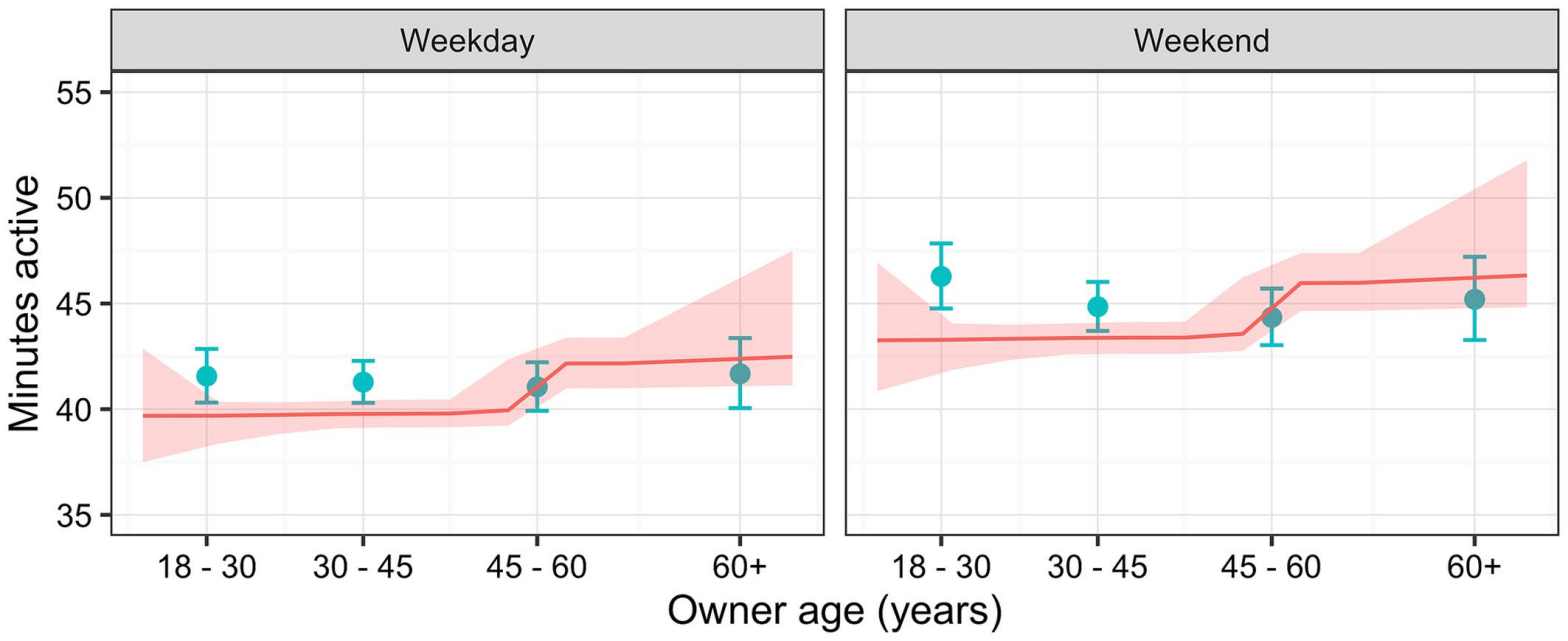
Relationship between active minutes as a function of owner age for each day type (weekday or weekend), whilst holding all other relevant variables (season, climate, location and latitude) constant, estimated using a linear mixed-effects (LM; blue) and a Bayesian additive regression trees (BART; red) models. The LM predictions (blue) are for categories of owner age, where the points represent the median age for the category, and error bars represent 95% confidence intervals. The BART model predictions (red line) depict continuous values for owner age, with the ribbon depicting the 95% credible interval.

Dogs were more active on weekend days [44.7 active minutes (44.3, 45.1)] than on weekdays [41.3 active minutes (41.0, 41.7); *p* < 0.001], whilst those living in rural locations [43.6 active minutes (43.1, 44.1)] were slightly more active than those in urban areas [42.4 active minutes (42.0, 42.8); *p* < 0.001]. Finally, compared with the 18–30 years reference [44.6 active minutes (44.1, 45.1)], dogs whose owners were 30–45y [42.8 active minutes (42.4, 43.2)], 45–60y [42.7 active minutes (42.3, 43.2)] or ≥60y [41.9 active minutes (41.3, 42.5)] were less active (*p* < 0.001). Figure 6 shows the predicted activity varying with both owner age and day type, where dogs of owners <60y were more active on weekends than weekdays, whilst activity was similar whatever the day type in dogs whose owners were ≥60y.

## 4 Discussion

The aim of this study was to examine associations between physical activity and both dog and environmental characteristics, using data from a large population of domestic dogs living in the USA utilizing a large and novel dataset of accelerometer data and EHRs. We were able to estimate typical activity patterns of pet dogs with differing characteristics living in a range of different environments. It is hoped that such large-scale estimates of dog activity, at the population-level, could be used to inform daily exercise and caloric requirements for dogs, across a range of different settings. The study design combined a population-level epidemiological analysis, utilizing EHRs from >1,000 veterinary hospitals, with objective physical activity recording at an individual level by accelerometer. Therefore, this analysis provides a unique insight into the activity levels of pet dogs living in different environments, enabling the effects of many different animal and environmental variables to be studied. Previous research on dog activity has been based on either small accelerometer-based datasets (19, 21, 22, 38, 69), which may not generalize at the population-level, or owner survey data (9, 70–72), which may be subjective. Given that actual movement is recorded, accelerometers can more accurately measure physical activity than subjective approaches such as owner surveys. In previous research on human adults, agreement between reported activity estimates and accelerometer measurements was poor (73), which is also likely to be the case also for estimates of dog activity. For example, in one study, owners were asked to estimate how active their dog was (9) with the average being 2.4 h per day, which is markedly greater than estimates of recorded activity in the current study (51 active minutes per day).

The dog variable most strongly associated with physical activity was dog age where, not surprisingly, younger dogs were far more active than older dogs. Physical activity also varied with dog size, whereby larger breeds of dog were more active, which is supported by previous findings (7, 70). Exercise requirements are usually defined by breed (strongly linked to size), based on the average energy levels and exercise motivations of each breed (6, 70). However, there was also an interaction between dog age and breed size, with the greatest age-related decline seen in larger breeds of dog, especially those of the giant breeds. Although the reasons for this are not known, it might be related to a greater prevalence of orthopedic diseases, such as osteoarthritis, in larger breeds of dog, the incidence of which increases with age (74). However, given that lifespan varies by dog size (75), from an aging perspective, a 10-year-old small-breed dog is biologically younger than a large-breed dog of the same age.

In this study, there was no significant association between neuter status and activity levels. The effect of sex was marginally greater whereby, compared with female dogs, recorded physical activity in male dogs was greater by approximately 2 active minutes per day, on average. These findings for neutering and sex are similar to previous research, where both significant and non-significant associations have been seen for both relationships (7, 76, 77).

Previous studies exploring the relationship between physical activity and BCS in dogs have been inconclusive, with some indicating that dogs with a BCS in the overweight range are less active than those with a BCS in the optimal range (28, 76), and other studies not reporting such an association (11, 30). In the current study, no significant relationship between BCS and physical activity was observed. However, a limitation of the current study was the fact that the DAG might not have accurately depicted interactions between BCS and either illness or injury. In this respect, we assumed that BCS would have an effect on illness and injury, rather than the other way round, not least given the evidence of associations between obesity and various illnesses, including osteoarthritis, cardiorespiratory function, neoplasia, diabetes mellitus, cruciate ligament disease, condylar fractures and disc disease (78–82). However, effects might also occur in the opposite direction, whereby some illnesses and injuries could affect weight status unrelated to physical activity; for example, chronic kidney disease (83), neoplasia (84) and diabetes mellitus (85) can also lead to weight loss and loss of body condition; since they might also negatively affect physical activity, it will tend to oppose the effect of overweight condition on physical activity. A limitation of the measurement approach was that we only studied minutes active and not the intensity of activity (which is also measured by the activity monitors), and this might have differed more by weight status. We also did not consider the possible impact of dogs undergoing weight management; in this respect, owners might have increased physical activity as part of controlled weight reduction protocol as this is commonly recommended (86).

This study also explored the associations between environmental attributes and physical activity. Dogs living in hot-dry or marine climates were more active than those living in cold or hot-humid climates. Such associations are probably related to either challenges with exercising, both for the owner and dog, when weather conditions are more extreme, for example very hot or cold weather. Recorded activity was also marginally greater in dogs living in rural areas compared with those in urban areas, which could be due to availability of spaces for walking or the size of yards to enable dogs to exercise at home. It might also be because the number of working dogs was greater in rural areas.

Much of the physical activity undertaken by dogs occurs in the presence of their owners and includes walking and other interactions (87). Not surprisingly, therefore, more activity was recorded at weekends, when owners are more likely to be available, than on weekdays. Physical activity also varied with owner age whereby most active minutes were recorded in dogs whose owners were either aged between 18 and 30 years or were 60 plus. Although the reasons for this are not clear, it might be the result of complex interactions between variables such as physical fitness (likely to be greater in younger adults) and both work and family commitments including parenthood (likely to be greatest in adults between 30 and 60). Besides these simple associations with day of the week and owner age, there was also an interaction between the two, whereby the difference between weekday and weekend activity decreased as owner age increased. Again, this could be the result of difference in availability of exercise at different time due to work or family commitments; most notably, the difference between weekday and weekend activity was least pronounced for the 60 + age group, which is likely due to many owners in that age group being retired.

On average, dogs recorded 51 active minutes per day, although the exact amount varied widely from less than 10 to over 600 min. If representative of the general dog population, these data might help to inform population-based recommendations on activity and, in particular, food requirements. Most notably, given the wide distribution in amounts of physical activity, single population-level recommendations would be problematic and, instead, a degree of tailoring based on dog and environmental characteristics would be sensible. For example, reductions in food intake could be considered during seasons where dogs are less active (e.g., winter and summer), for dogs in hot humid climates, those in urban areas and also on weekdays; further, adjustments could also be considered based on age (less for older dogs), breed (less for giant breeds) and sex (less for female dogs). For dogs routinely wearing an activity monitor, it might also be feasible to tailor food portions to daily activity counts. Of course, given the limitations in technology and accuracy, further validatory work would be required before such an approach could be recommended. It may be that food intake is best optimized to the individual, based on response of bodyweight to a known amount of food. Such a trial-and-error approach could be used by pet owners, guided by veterinary professionals.

This study utilized a DAG to identify the relevant adjustment sets for modeling the associations between activity and dog and environmental attributes using linear regression. This technique is becoming more popular within human healthcare research (48) but is not commonly used in dog health research. The technique has several advantages, for example, helping to guide statistical analysis by ensuring that appropriate variables are included, and also flagging possible biasing effects in model creation (42, 43, 45). A limitation of this approach is that any DAG is only as good as the variables that are included: some important effects might have been overlooked, either because they are unknown or because data on the variable is not available. However, this is equally likely in traditional associational linear modeling, and the construction of the DAG and validation with data help understand the likelihood of missing or unknown variables within the process.

In light of the limitations with our approach of linear modeling using variables identified by the DAG, we employed a second novel technique for validation, creating BART models. This statistical method is relatively new within both human healthcare and dog health research but has the advantage of fitting the data in a non-parametric and non-linear manner; this allows potentially non-linear relationships between attributes to be fitted more accurately and flexibly than with a linear methods, whilst avoiding overfitting as a result of Bayesian priors. Such BART models have proved to be successful in causal inference competitions (88). In a similar manner to linear modeling, results generated by this method can be interpreted using marginal means and partial dependence plots, although their non-parametric nature precludes them from producing coefficient estimates. Nonetheless, where the BART model supports the linear model, more confidence may be given to the estimates produced; in this respect, the fact that an inductive approach (BART) produced similar results to a deductive approach (linear modeling), would suggest that the adjustment set used was correct and, of course, would also support the underlying DAG as a representation of the real-world. In contrast, disagreement between the models would indicate a poor fit of the linear model, either due to misspecification or limitations of linear modeling approaches. In future, the results of the BART model could be used to guide the specification of the linear model; however, for this work, the BART model was used as a validation approach and the results were not iterated back to the linear model.

As always, study limitations should be considered. First, study data were collected from the Pet Insight Project, which involved the distribution of 100,000 free activity monitors to Banfield clients. Since this was a voluntary process, there may have been bias in the owners who engaged toward those interested in physical activity and fitness, or more conscientious dog owners. Further, long-term use of an activity monitor might be more likely in dogs that are already more active, for example younger dogs, whilst owners of older dogs with health issues might also be interested in monitoring their activity. Very small dogs (such as Chihuahuas) may be less likely to wear activity monitors, which is supported by the comparison of the population of pets visiting Banfield Pet Hospitals and those wearing Whistle monitors. Further, there might have been differences in activity for working dogs and competition dogs (e.g., those engaging in agility trials) but, since there was no way of identifying them from the records, this group could not be studied specifically. However, the population selected for this study was similar to the general population attending the veterinary hospital network, in terms of proportions of dogs and visits of each breed size. Further, for each breed size group, the average age at a visit for dogs in the study was within one standard deviation of the veterinary hospital population. These findings imply that the influence of possible selection bias in the study population was limited.

A second limitation was the fact that we did not attempt to assess different types of activity separately, for example walking versus running. Although the activity monitors used do categorize activity into different categories, given that more complex algorithms are required for these endpoints, we were concerned that there would be greater variability and inaccuracies within the data. Therefore, we instead chose to focus on total activity above a particular threshold, to ensure consistency in data measurement. A third limitation was the fact that the study utilized a DAG to explore possible relationships and potential biases among various dog and environment attributes and how they might impact physical activity. The approach was evidence based because the attributes included in the DAG were based on previous research findings (Supplementary information). However, we might have missed some variables influencing physical activity, due to lack of awareness. Further, the assumption of a unidirectional associations among variable could also be problematic, as discussed above for associations between weight status and the presence of illness or injury. Nonetheless, the fact that there was consistency between the results identified with two different statistical approaches, non-parametric inductive BART model and the linear model, partially mitigates this concern. Further, the impact of treating various medical conditions was not considered. Finally, we did not specifically examine the impact of neoplastic diseases on activity, not least given that they have a wide spectrum of severity, from benign with minimal health impact (e.g., small lipoma not affecting movement) to severe with a major health impact (e.g., malignant, metastatic disease process). However, by using the “unhealthy visit” tag for EHRs, neoplastic diseases, and indeed those caused by other etiologies, could be identified. Nonetheless, given that no checks were performed to confirm the reliability of this approach, we cannot be certain that all dogs with illnesses affecting physical activity were identified, potentially impacting on the accuracy of estimates from our statistical models.

## 5 Conclusion

In conclusion, we have used accelerometer-derived activity data gathered from pet dogs living in North America, to determine associations with both dog and environmental characteristics. Age was the dog characteristic most strongly associated with the number of daily active minutes, whilst associated environmental variables included climate, season, location (urban vs. rural) and day of the week (weekday vs. weekend). Knowledge of these associations could be used to inform daily exercise and caloric requirements for dogs, and how they should be adapted according to individual circumstances. The wide variation in activity levels suggest population-level recommendations, particularly for feeding guides, may be unsuitable, and more individualization is required.

## Data Availability

The data analyzed in this study is subject to the following licenses/restrictions: Electronic health record and activity data is proprietary and contains unique patient identifiers so public access would compromise client/patient confidentiality. Additional data has been obtained from a third party. Requests to access these datasets should be directed to abigail.orourke@effem.com.
