# Supplementary material for: Estimated activity levels in dogs at population scale with linear and causal modeling
Source: Front Vet Sci. 2025 Jul 10;12:1572794. doi: 10.3389/fvets.2025.1572794 (PMC12287635; doi:10.3389/fvets.2025.1572794)
Supplement: Supplementary file 1 [file Table_1.docx]

Supplementary Material

# Supplementary Table 1

Detail of hypotheses supporting the directed acyclic graph (DAG) constructed to capture knowledge and assumptions related to dog activity. Each node (variable) and edges (effects) are described. The data referred to are electronic health records (EHR) and daily accelerometer activity records.

| **Node** | **Description** |
| --- | --- |
| **Age:** recorded as the time span between the date of birth recorded in the EHR and the data of the activity record. | |
| Activity | It is expected that activity will decrease with age (D. C. Brown et al., 2010; Lee et al., 2022; Morrison et al., 2014; Woods et al., 2020). |
| Health | Many diseases are age-related, so health status is expected to depend on age (Anderson et al., 2018). |
| Injury | Dogs may be more likely to be injured at specific ages, for example young dogs and old dogs. |
| Body condition score | It is expected that as a dog ages, it is more likely to become overweight. |
| Neuter status | Dogs tend to be neutered in their first few years of life, hence an older dog is more likely to be neutered than a younger dog. |
| **Size:** Size is a categorical static attribute that is based on the average size category of the breed of dog determined from the EHR data. If the dog has no reported breed, its size is estimated from its mean BCS-corrected weight over the ages 2-10 (i.e., one weight value converted to one size value for a specific pet). Size categories are toy, small, medium-small, medium-large, large and giant. | |
| Activity | It is expected that dogs of different size categories may display different activity levels, for instance large dogs may be more active than toy dogs (Morrison et al., 2014; Pickup et al., 2017). |
| Health | Prevalence of health conditions varies by breed (and therefore approximately size) of dogs (D. Brown et al., 1996; Graves et al., 2023). |
| Injury | This edge was included based on a conditional dependency identified through validating the DAG against correlations in the data (D. Brown et al., 1996; Graves et al., 2023). Toy dogs appear to have higher rates of injury than other sizes when confounders are held constant. |
| Body condition score | BCS may be dependent on size (via breed), as specific breeds are more likely to be overweight (e.g., pugs, Labradors). |
| Neuter status | Size of dog may impact the decision to neuter; larger dogs may be more likely to be neutered than smaller dogs. |
| **Health:** Health status is a categorical attribute, where “True” indicates that the pet has a health condition recorded on its EHR that may impact activity, otherwise “False”. The health status must have been recorded on the EHR prior to the activity record. Unhealthy ailments terms, using Banfield terms, are *arthritis, osteoarthritis, joint disease, degenerative joint disease, spondylosis, geriatric pet, hepatopathy, chronic renal failure, ovariohysterectomy, castration, osteosarcoma, murmur, brachycephalic syndrome, malaise, medical intervert disc dis, tracheal collapse, generalised muscle atrophy*. Additionally, any ailments falling under the Banfield-defined category of neurology, endocrinology and cardiovascular system are considered unhealthy. These ailments were selected based on frequency of diagnosis and potential to impact activity. | |
| Activity | Health status is expected to have an impact on activity as certain conditions may reduce activity levels. |
| **Injury:** Injury status is a categorical attribute, where “True” indicates that the pet has a injury-type diagnosis on its EHR that may impact activity, otherwise “False”. The injury must have been recorded on the EHR prior to the activity record. Banfield terms are patellar luxation, hip joint luxation, lameness, muscle atrophy, sprain, strain, muscle trauma, hip dysplasia, cruciate ligament, hit by car, medical soft tissue injury, surgical foreign body, gunshot wound, foreign body (gastrointestinal tract), hernia, toenail injury. | |
| Activity | Injury status is expected to have an impact on activity as certain conditions may reduce activity levels. |
| **Body conditions score:** Body condition score is a categorical attribute taken from the EHRs. It is aggregated into ideal + underweight and overweight + obese; essentially a flag is created to indicate if the pet is overweight or not. BCS is taken from EHRs recorded prior to the activity record. | |
| Activity | It is hypothesised that pets that are overweight may do less activity, either due to exercise motivation (being overweight making exercise more difficult) or due to lifestyle impacting both (low exercise lifestyle leads to less historical activity leads to increased current BCS) (Chapman et al., 2019; Griss et al., 2021; Morrison et al., 2013, 2014). Note that this historical lifestyle factor cannot be accounted for in this analysis. |
| Health | Overweight pets may be more likely to have health issues (Bach et al., 2007; D. Brown et al., 1996; Graves et al., 2023; Lund et al., 2006; Partington et al., 2022). This mechanism may also work the other way, in that certain health conditions may impact BCS. However, the former direction is more likely and more supported. Given that BCS is slow to change, and health status is recorded at the visit, it is likely that BCS state precedes health condition in the timeline, and so BCS should impact health condition and not vice versa, for this analysis. |
| Injury | Overweight pets may be more likely to become injured due to increased weight causing increased load on limb structures. |
| **Sex:** Sex is a static categorical attribute taken from the EHRs. | |
| Activity | It is hypothesised that male pets may be more active than female pets (Griss et al., 2021; Li et al., 2022; Morrison et al., 2014). |
| Body condition score | It is hypothesised that female pets may have higher BCS than male pets. |
| Neuter status | Probability to neuter a pet may depend on the sex of the pet. |
| **Neuter status:** Neuter status is a categorical attribute taken from the EHRs. A pet may change neuter status once over the course of its records (go from entire to neutered). The neuter status at activity record is determined by comparing the date of the activity record to the date of neutering, if recorded. If no date is defined, but the status is recorded as neutered in the EHR, the pet is assumed to be neutered at all activity records. | |
| Activity | Entire pets are thought to be more active than neutered pets (Griss et al., 2021; Li et al., 2022; Morrison et al., 2014). |
| Health | The probability of some health conditions is altered due to neutering. |
| **Climate:** Climate is a static categorical attribute determined from the county and state given in the owner data combined with the climate regions defined in the Building America project. | |
| Activity | It is possible that dogs are less active in extreme climates. |
| Size | Climate may impact breed which may be represented in size. |
| **Latitude:** Latitude is a static attribute taken from the owner data directly and is use as a numeric value. | |
| Activity | In northern latitudes, daylight hours may limit activity levels. |
| Climate | Northern latitudes are more likely to have cold climates, southern latitudes are more likely to have hot climates. |
| **Season:** Season is derived from the date of the activity record and is categorical (spring, summer, autumn, winter). | |
| Activity | Seasons such as summer and winter may reduce activity levels due to extreme weather conditions or limited daylight hours. |
| **Day:** Day is a categorical variable of “weekend” or “weekday”, derived from the date of the activity record. | |
| Activity | Higher activity levels may be expected on a weekend, due to increased owner availability (Piccione et al., 2013; Potter et al., 2020). |
| **Location:** Location is a static categorical variable of either “Urban” or “Rural”. Location is derived from the owner data based on address. | |
| Activity | Dogs in rural areas may be more active than those in urban areas, either due to availability of green spaces or higher population of working dogs in rural areas (Lee et al., 2022; Piccione et al., 2013). |
| Size | Urban areas may have higher populations of smaller dogs due to the environment (smaller housing or gardens). |
| **Owner age:** Owner age is recorded as the time span between the date of birth recorded in the owner data and the date of the activity record. | |
| Activity | Availability and motivation to exercise may be dependent on owner age (Lee et al., 2022). |
| Age | Older owners may have older pets. |
| Size | Choice of size of pet may vary with age, such as older owners preferring smaller pets. |
| Body condition score | Unrecorded environmental factors may depend on owner age and may impact body condition score, such as feeding routines. |

**References for Supplementary Table 1**

Anderson, K. L., O’Neill, D. G., Brodbelt, D. C., Church, D. B., Meeson, R. L., Sargan, D., Summers, J. F., Zulch, H., & Collins, L. M. (2018). Prevalence, duration and risk factors for appendicular osteoarthritis in a UK dog population under primary veterinary care. Scientific Reports, 8(1), 5641. https://doi.org/10.1038/s41598-018-23940-z

Bach, J. F., Rozanski, E. A., Bedenice, D., Chan, D. L., Freeman, L. M., Lofgren, J. L. S., Oura, T. J., & Hoffman, A. M. (2007). Association of expiratory airway dysfunction with marked obesity in healthy adult dogs. American Journal of Veterinary Research, 68(6), 670–675. https://doi.org/10.2460/ajvr.68.6.670

Brown, D. C., Michel, K. E., Love, M., & Dow, C. (2010). Evaluation of the effect of signalment and body conformation on activity monitoring in companion dogs. American Journal of Veterinary Research, 71(3), 322–325. https://doi.org/10.2460/AJVR.71.3.322

Brown, D., Conzemius, M., & Shofer, F. (1996). Body weight as a predisposing factor for humeral condylar fractures, cranial cruciate rupture and intervertebral disc disease in Cocker Spaniels. Veterinary and Comparative Orthopaedics and Traumatology, 8, 75–78.

Chapman, M., Woods, G. R. T., Ladha, C., Westgarth, C., & German, A. J. (2019). An open-label randomised clinical trial to compare the efficacy of dietary caloric restriction and physical activity for weight loss in overweight pet dogs. The Veterinary Journal, 243, 65–73. https://doi.org/https://doi.org/10.1016/j.tvjl.2018.11.013

Graves, J. L., McKenzie, B. A., Koch, Z., Naka, A., Spofford, N., & Morrison, J. (2023). Body weight, gonadectomy, and other risk factors for diagnosis of osteoarthritis in companion dogs. Frontiers in Veterinary Science, 10. https://doi.org/10.3389/fvets.2023.1275964

Griss, S., Riemer, S., Warembourg, C., Sousa, F. M., Wera, E., Berger-Gonzalez, M., Alvarez, D., Bulu, P. M., Hernández, A. L., Roquel, P., & Dürr, S. (2021). If they could choose: How would dogs spend their days? Activity patterns in four populations of domestic dogs. Applied Animal Behaviour Science, 243. https://doi.org/10.1016/j.applanim.2021.105449

Lee, H., Collins, D., Creevy, K. E., & Promislow, D. E. L. (2022). Age and Physical Activity Levels in Companion Dogs: Results From the Dog Aging Project. Journals of Gerontology - Series A Biological Sciences and Medical Sciences, 77(10), 1986–1993. https://doi.org/10.1093/gerona/glac099

Li, M. F., Nagendran, L., Schroeder, L., & Samson, D. R. (2022). The activity patterns of nonworking and working sled dogs. Scientific Reports, 12(1). https://doi.org/10.1038/s41598-022-11635-5

Lund, E., Armstrong, J., Kirk, C., & Klausner, J. S. (2006). Prevalence and risk factors for obesity in adult dogs from private US veterinary practices. Intern J Appl Res Vet Med, 4, 177–186.

Morrison, R., Penpraze, V., Beber, A., Reilly, J. J., & Yam, P. S. (2013). Associations between obesity and physical activity in dogs: A preliminary investigation. Journal of Small Animal Practice, 54(11), 570–574. https://doi.org/10.1111/jsap.12142

Morrison, R., Penpraze, V., Greening, R., Underwood, T., Reilly, J. J., & Yam, P. S. (2014). Correlates of objectively measured physical activity in dogs. Veterinary Journal (London, England : 1997), 199(2), 263–267. https://doi.org/10.1016/J.TVJL.2013.11.023

Partington, C., Hodgkiss-Geere, H., Woods, G. R. T., Dukes-McEwan, J., Flanagan, J., Biourge, V., & German, A. J. (2022). The effect of obesity and subsequent weight reduction on cardiac structure and function in dogs. BMC Veterinary Research, 18(1), 351. https://doi.org/10.1186/s12917-022-03449-4

Piccione, G., Arfuso, F., Giannetto, C., Faggio, C., & Panzera, M. (2013). Effect of housing conditions and owner’s schedule on daily total locomotor activity in dogs (Canis familiaris). Biological Rhythm Research, 44(5), 778–786. https://doi.org/10.1080/09291016.2012.756254

Pickup, E., German, A. J., Blackwell, E., Evans, M., & Westgarth, C. (2017). Variation in activity levels amongst dogs of different breeds: Results of a large online survey of dog owners from the UK. Journal of Nutritional Science, 6. https://doi.org/10.1017/jns.2017.7

Potter, K., Marcotte, R., Petrucci Jr, G., Rajala, C., Linder, D., & Balzer, L. (2020). Examining the Contribution of Dog Walking to Total Daily Physical Activity Among Dogs and Their Owners. Journal for the Measurement of Physical Behaviour, 4, 1–5. https://doi.org/10.1123/jmpb.2020-0059

Woods, H. J., Li, M. F., Patel, U. A., Lascelles, B. D. X., Samson, D. R., & Gruen, M. E. (2020). A functional linear modeling approach to sleep–wake cycles in dogs. Scientific Reports, 10(1). https://doi.org/10.1038/s41598-020-79274-2

# Supplementary Table 2

Counts of dogs, stratified by breed, within the Banfield, study (all visits) and Whistle populations.

| **Breed ^1^** | **Banfield population ^2^** | **Study population** | **Whistle population^3^** |
| --- | --- | --- | --- |
| Alaskan Husky Mix | - | - | 1,097 (0.6%) |
| American Staffordshire Terrier | 194,263 (4.0%) | 805 (2.8%) | 6,214 (3.5%) |
| American Staffordshire Terrier Mix | 95,768 (2.0%) | 586 (2.1%) | 2,537 (1.4%) |
| Australian Cattle Dog | 24,972 (0.5%) | 175 (0.6%) | 1,174 (0.7%) |
| Australian Cattle Dog Mix | 23,092 (0.5%) | 204 (0.7%) | 1,804 (1.0%) |
| Australian Shepherd | 56,897 (1.2%) | 417 (1.5%) | 2,836 (1.6%) |
| Australian Shepherd Mix | 31,916 (0.7%) | 235 (0.8%) | 1,363 (0.8%) |
| Beagle | 58,673 (1.2%) | 464 (1.6%) | 3,006 (1.7%) |
| Beagle Mix | 43,506 (0.9%) | 397 (1.4%) | 1,872 (1.1%) |
| Border Collie | 22,393 (0.5%) | 189 (0.7%) | 1,665 (0.9%) |
| Border Collie Mix | 27,533 (0.6%) | 242 (0.9%) | 1,806 (1.0%) |
| Boxer | 74,036 (1.5%) | 421 (1.5%) | 1,219 (0.7%) |
| Boxer Mix | 43,589 (0.9%) | 332 (1.2%) | 1,276 (0.7%) |
| Chihuahua | 240,004 (4.9%) | 719 (2.5%) | 872 (0.5%) |
| Chihuahua Mix | 154,412 (3.3%) | 741 (2.6%) | 2,640 (1.5%) |
| Dachshund | 92,269 (1.9%) | 435 (1.5%) | 1,138 (0.6%) |
| Dachshund Mix | 36,222 (0.7%) | 270 (1.0%) | 904 (0.5%) |
| English Bulldog | 51,159 (1.0%) | 195 (0.7%) | 228 (0.1%) |
| German Pointer | 10,704 (0.2%) | 93 (0.3%) | 1,574 (0.9%) |
| German Shepherd | 129,677 (2.7%) | 753 (2.6%) | 4,929 (2.8%) |
| German Shepherd Mix | 75,819 (1.6%) | 589 (2.1%) | 4,037 (2.3%) |
| Golden Retriever | 96,192 (2.0%) | 606 (2.1%) | 5,438 (3.1%) |
| Golden Retriever Mix | 39,296 (0.8%) | 290 (1.0%) | 816 (0.5%) |
| Great Pyrenees | 10,021 (0.2%) | 107 (0.4%) | 1,560 (0.9%) |
| Great Pyrenees Mix | 6,338 (0.1%) | 87 (0.3%) | 1,090 (0.6%) |
| Labrador Retriever | 227,285 (4.6%) | 1,564 (5.5%) | 9,082 (5.1%) |
| Labrador Retriever Mix | 214,527 (4.4%) | 1,731 (6.1%) | 1,342 (0.8%) |
| Maltese | 83,031 (1.7%) | 224 (0.8%) | 391 (0.2%) |
| Maltese Mix | 86,744 (1.8%) | 392 (1.4%) | 546 (0.3%) |
| Miniature Schnauzer | 50,060 (1.0%) | 301 (1.1%) | 1,074 (0.6%) |
| Pomeranian | 65,923 (1.3%) | 210 (0.7%) | 569 (0.3%) |
| Poodle | 50,120 (1.0%) | 142 (0.5%) | 904 (0.5%) |
| Poodle Mix | 91,128 (1.9%) | 444 (1.6%) | 2,467 (1.4%) |
| Pug | 52,879 (1.1%) | 223 (0.8%) | 570 (0.3%) |
| Shiba Inu | 17,257 (0.4%) | 159 (0.6%) | 1,351 (0.8%) |
| Shih Tzu | 187,746 (3.8%) | 586 (2.1%) | 1,066 (0.6%) |
| Shih Tzu Mix | 78,046 (1.6%) | 357 (1.3%) | 632 (0.4%) |
| Siberian Husky | 86,708 (1.8%) | 749 (2.6%) | 6,073 (3.4%) |
| Siberian Husky Mix | 34,050 (0.7%) | 260 (0.9%) | 1,860 (1.0%) |
| Yorkshire Terrier | 197,683 (4.0%) | 716 (2.5%) | 1,163 (0.7%) |
| Yorkshire Terrier Mix | 68,021 (1.4%) | 349 (1.2%) | 477 (0.3%) |
| Mixed Breed | 386,081 (7.9%) | 2,312 (8.1%) | 3,311 (1.9%) |
| Other | 1,285,746 (26.2%) | 8,491 (29.8%) | 71,191 (39.9%) |

Data are reported as number (%). ^1^ The top 30 breeds in each population are listed separately, with remaining breeds aggregated into the “Other” category. ^2^ The Banfield population is defined as dogs visiting Banfield Pet Hospitals between in the years 2013 to 2023, to align with the data collection period for the study. ^3^ The Whistle population is defined as dogs with a Whistle device between the years 2013 to 2023, to align with the data collection period for the study. ^4^ The “Alaskan Husky” breed does not exist in the Banfield database and, therefore, numbers for the Banfield and study populations are not available.
